# New Water Oxidation Mechanism in Photosystem II Resolves Major Experimental Controversies

**DOI:** 10.1002/anie.1930324

**Published:** 2026-06-22

**Authors:** Yulia Pushkar

**Affiliations:** ^1^ Department of Physics and Astronomy Purdue University West Lafayette Indiana USA

**Keywords:** DFT, H‐bond, Kok cycle, Mn_4_Ca cluster, O─O bond formation mechanism, oxygen evolving complex, photosystem II

## Abstract

Light‐driven oxygen formation in photosystem II protein is a fundamental process that sustains our biosphere. The research by advanced physical techniques has delivered new insights on the structure and function of the Mn_4_CaO_5_ cluster. However, discrepancies in experimental observations and computational models persist, impeding the understanding of O–O bond formation and the role of the protein environment in the process. Here, we show that i) assignment of the OEC unique oxygen O3 ligated by histidine (His337) via dynamic H‐bond as a slow exchanging substrate and ii) its coupling with O6 oxygen generated at Mn1 in the S_2_ to S_3_ transition—giving an O─O bond formation mechanism most consistent with all currently available experimental data. We propose a mechanism by which the protein environment can steer the O─O bond formation by charge control via H‐bonding and open coordination of Mn1. Obtained O3–O6 peroxide is at lower energy than peroxides in the most studied O5─O6 bond formation pathway. His337 appears to be similar to distal His in globins used for management of the O_2_ and H_2_O_2_ intermediates. This new mechanism breaks the prior impasse and will undoubtedly invigorate future detailed studies uncovering further details.

## Graphical abstract

In Photosystem II Oxygen Evolving Complex, we discovered the O3‐O6 peroxide at lower energy. Assignment of the O3 ligated by histidine (His337) as a slow exchanging substrate and its coupling with O6 give the O─O bond formation mechanism most consistent with all currently available experimental data. Proposal shows how protein environment can steer the O─O bond formation by charge control via H‐bond and open coordination of Mn1. The new mechanism breaks the prior impasse and will invigorate future detailed studies.

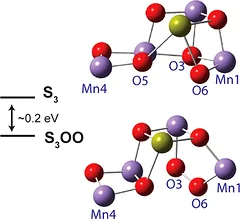

## 1 Introduction

**FIGURE 1:**
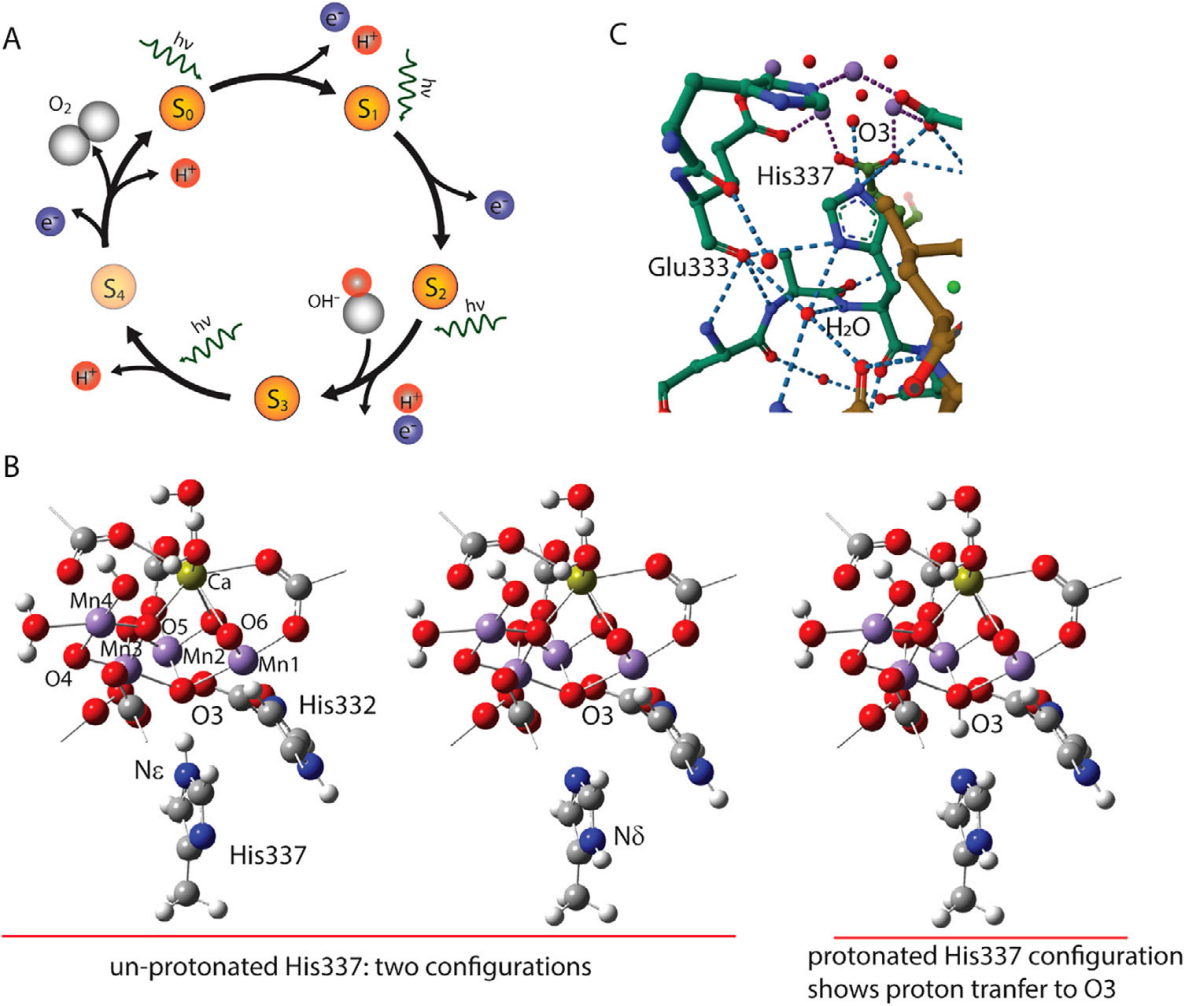
(A) Scheme for the Kok cycle showing light‐induced electron transfer events and definition of the S‐state intermediates. (B) Model of the oxygen evolving complex of the photosystem II in the S_3_‐state. Four Mn and one Ca^2+^ ions are shown with six bridging carboxylate ligands and two histidine residues. Side chains are truncated to provide clear view. Interaction between the His337 and O3 oxygen are analyzed as having three possible configurations where i) O3 is protonated (O3H) as this configuration is relevant for slow substrate exchange); ii) in H‐bonding with N‐H group; and iii) lacks H‐bond—a state which lowers the energy formation of O3–O6 peroxide. (C) Visualization (from 8IR5) of the His337‐ Nδ nitrogen environment. Nδ can form H‐bond with backbone of Glu333 and conserved water molecule, which is involved with extended H‐bonding network.

Photosystem II (PSII) is a membrane‐bound protein‐pigment complex found in plants, algae, and cyanobacteria that catalyzes the light‐driven oxidation of water to molecular oxygen, thereby sustaining the aerobic life on Earth. At the heart of the light‐induced water oxidation is the oxygen‐evolving complex (OEC), a catalytic Mn_4_CaO_5_ cluster embedded within the protein environment [1, 2, 3, 4, 5]. The cluster has an intricate ligand environment with a total of six carboxylates bridging its metal ions: three are bridging Mn–Mn pairs and three are bridging the Mn–Ca pairs [4, 6]. One His332 directly coordinates Mn1 via a Mn─N bond and one His337 provides the hydrogen bond to a bridging oxygen atom O3, Figure 1. Ca and Mn4 are also coordinated with OH*_x_* fragments, which are most probably both waters on Ca^2+^ and can be a combination of waters and ─OH for Mn4. Protonation states of all bridging oxygens are not known with certainty and can depend on the S‐state of the Kok cycle, Figure 1A. The Kok cycle serves as a framework for understanding the overall OEC function as it explains how 4 light‐induced electron‐transfer events couple to the four‐electron (2H_2_O → 4H^+^+O_2_+4e^−^) water oxidation reaction. Here, we will focus on more oxidized S_2_ and S_3_ states and pathways of O‐O bond formation in the S_3_ to S_0_ transition, forgoing the more‐reduced S_0_ and S_1_ states. The S_2_ state with a spin S = 1/2 configuration has provided a wealth of coordination and electronic structure information via EPR‐measured hyperfine splitting, such as ^1^H, ^14/15^N, ^55^Mn and ^17^O [7, 8]. While we have an abundance of structural information [4] and information about localization of the spin‐density (at least in the S_2_‐state), the mechanism of the O─O bond formation remains a topic of debate [9, 10, 11, 12, 13, 14]. This difficulty stems in part from the fact that the OEC has many oxygen atoms, and its environment also features water molecules that can potentially participate in O─O bond formation via a water nucleophilic attack mechanism.

The important analysis, which complements the OEC structural studies, is the study of the oxygen substrate exchange by time‐resolved isotope ratio membrane‐inlet mass spectrometry in combination with select spectroscopic techniques [7, 15, 16, 17, 18, 19]. The most significant substrate water exchange results can be summarized as follows. In the S_0_‐state, only one substrate water is observed to exchange at a rate of 10 s^−1^. In S_1_, the substrate water exchanges at a rate of only 0.02 s^−1^, and in S_2_ the rate increases to 2 s^−1^. In S_2_, a second substrate water is observed to exchange almost as fast as if it was not bound. In S_3_, there is a slow substrate water exchange (W_S_) at a rate of 2 s^−1^, and a fast exchange (W_f_) at 40 s^−1^. The two most interesting of these observations, both quite unexpected, are that the slow exchange is actually faster in S_2_ than in S_1_ and that the slow exchange is the same in S_2_ and S_3_. In addition, the Ca^2+^ cofactor substitution with Sr^2+^, either by chemical exchange or biosynthetic incorporation, enhances the rate of W_S_ but leaves W_f_ unchanged [7, 15, 18, 20]. This suggests that W_S_ is either associated with the Ca^2+^ site by acting as a bridge between the Mn and Ca, or that the process of substrate exchange involves transient coordination of Ws with both Mn and Ca.

In this manuscript, we deliver a new assignment of the fast and slow exchanging oxygens and a mechanism of O─O bond formation that follows from this new assignment. The outlined mechanism is currently the one most consistent with all available experimental data and resolves controversies of previous mechanisms that currently impede the progress in analysis of OEC and design of biomimetic assemblies.

## 2 Results and Discussion

### 2.1 Recent Assignment of O5 as Ws Faces Several Contradictions

The most recent detailed study performed parallel substrate water exchange experiments in the S_1_ state of native Ca‐PSII and biosynthetically substituted Sr‐PSII employing time‐resolved membrane inlet mass spectrometry (TR‐MIMS) and a time‐resolved ^17^O‐electron‐electron double resonance detected NMR (TR‐^17^O‐EDNMR) approach [7]. Authors interpreted results as a correlation of an oxygen bridge between the Mn3 and Mn4 ion (O5) exchange with the same rate as a rate of exchange as the slowly exchanging substrate water (W_S_) in the TR‐MIMS experiments and ii) that the exchange rates of O5 and W_S_ are both enhanced by Ca^2+^→Sr^2+^ substitution in a similar manner. The conclusion was made that only O5 fulfills all criteria for being W_S_ [7]. Some deliberations were also presented as to exclude the water ligand on Mn4 as being not suitable for W_s_ assignment.

However, the O5 is a bridging oxygen in a di‐µ‐oxo Mn4–Mn3 unit, which is not protonated. Such bridging oxygens between high valent Mn ions exchange extremely slowly (<10^−2^ s^−1^ for Mn_2_(III, IV) and <10^−6^ s^−1^ for Mn_2_(IV, IV)). In addition, this bridging oxygen being deprotonated means that its binding energy to a metal ion should increase and thus decrease its exchange rate strongly. This first contradiction has been explained away with detailed DFT calculations; however, the feature of the back electron transfer from the S_3_ state to Tyr_Z_ was required to show that exchange is still possible in the S_3_ state [21]. Calculations also featured water coordination to an otherwise five‐coordinated Mn1 with subsequent protonation of the O5 and transition to closed cubane conformation necessary in all S_1_, S_2_ and S_3_ state exchange reaction schemes [21]. Guo et al. repeated some of these calculations later, essentially reproducing the main results and emphasizing once again the requirement of open—closed cubane interconversion to model Ws water exchange at O5 [22]. Currently, closed cubane remains to be an experimentally unconfirmed geometric state in the OEC [23, 24].

The second contradiction is that the measured hyperfine splitting (hfs) associated with ^17^O do not exceed ∼8 MHz [7], which is extremely low for bridging oxygen. It is well‐known that in the OEC there is a significant hole delocalization onto the bridging oxygens of the di‐µ‐oxo Mn‐Mn units such as O5. Thus, ^17^O hfs might reach, at least theoretically, as high as 60–50 MHz on some of their components, Table S1. Table S1 shows ^17^O hfs computed for several DFT models of the S_2_ state, *S* = 1/2. Some of these models were reported earlier [24, 25], and others are re‐optimizations with a different protonation states, Table S2. Note that water coordinated to Mn4 was predicted to have low hfs (Table S1) in agreement with earlier analysis concluding that it is unlikely to serve as a substrate oxygen. Interestingly the O3 came as the one candidate oxygen featuring “intermediate” hfs, likely due to H‐bonding with the His337. The pKa of His is ∼6, thus, at typical pH of PSII buffer solution, approximately 50% of His337 in a sample can be protonated. Table S2 compares DFT calculation of the S_2_ state model with protonated His337 and unprotonated His337. His337 protonation results in small ∼0.1 Å elongation of the Mn–Mn distances in the Mn3–Mn2 and Mn2–Mn1 bridges. Significantly, His protonation results in the changes in the H‐bonding pattern between the His337 and O3 where proton of the H‐bond between the O3—H‐His shifts to protonate O3: O3‐H—His. These results are similar to the computational analysis reported earlier [26, 27]. Interestingly, the His337 protonation was proposed to be responsible for S‐state dependent changes in polarized attenuated total reflectance Fourier transform infrared (ATR‐FTIR) spectroscopy [28], Quantum mechanics/molecular mechanics (QM/MM) calculations reproduced the characteristic changes of vibrational spectra and indicated that His337 remains protonated during the S‐state cycle ensuring high redox potential (Em value) of the Mn_4_CaO_5_ cluster throughout the reaction cycle to enable water oxidation [27, 28].

In the S_3_ model with Mn1 = O fragment, protonation of His results in similar elongations of Mn–Mn bonds of the open cubane core by ∼ 0.07–0.09 Å, Table S2; Figure 1B. O3 becomes protonated in the S_3_ state model with protonated His337. Thus, the pKa of this bridging oxygen is close to ∼6 in both analyzed S_2_ and S_3_ states showing that its protonation can be functionally relevant.

There were some proposals that O4 bridging Mn4 and Mn3 might also be protonated [12, 13, 14, 29] and function as a substrate due to the presence of an O4‐water channel, but such proposals were not confirmed from an energetic standpoint [30]. New synthetic Mn_4_CaO_4_ and Mn_4_SrO_4_ OEC models lack O4 bridging oxygen but form Mn_4_SrO_5_ under some solvent conditions potentially hinting at O4 been an active site [14, 31, 32]. In DFT calculations, the O4 was excluded as a possible substrate oxygen due to the unreasonably high energy of oxyl group formation at the O4 position [33]. O4 is adjacent to a water molecule (W539) and Arg357; however, the ∼3 Å O4‐to‐N (Arg357) distance precludes the formation of a strong H‐bond. The Arg pKa of ∼12 indicates that it will not donate the proton to the Mn‐O4‐Mn bridge at physiological conditions of pH∼6‐7. It was also shown that water cannot protonate this bridge [30]. The ^17^O hfs for O4 are as large as those for O5 due to either a lack of protonation or direct strong H‐bonding, Table S2.

Overall, the comprehensive analysis of the ^17^O hfs for the range of the S_2_‐state models in the *S* = 1/2 state has shown that O5 does not fit the experimentally measured ∼8 MHz signal. However, O3 is significantly more consistent with the properties of Ws from both EPR and substrate exchange properties, as will be shown below. His337 protonation further decreased ^17^O hfs of the O3 in the S_2_ state, Table S1. O3 is a frustrated oxygen placed in between four dissimilar positive charges: three Mn ions and one proton. Thus, O3 distances to Mn are longer than in a typical di‐µ‐oxo Mn–Mn unit, resulting in lower hole delocalization from Mn ions into O3, lower ^17^O hfs and a potentially lower binding energy that facilitates the O3 exchange on a sub‐seconds time scale. As we concluded that O3 is a more suitable oxygen center to explain the experimentally determined ∼8 MHz ^17^O hfs splitting [7], we continue to discuss how this center can meet the substrate exchange requirements imposed by experimental observations.

### 2.2 Plausibility of the O3 Assignment as Ws

**FIGURE 2:**
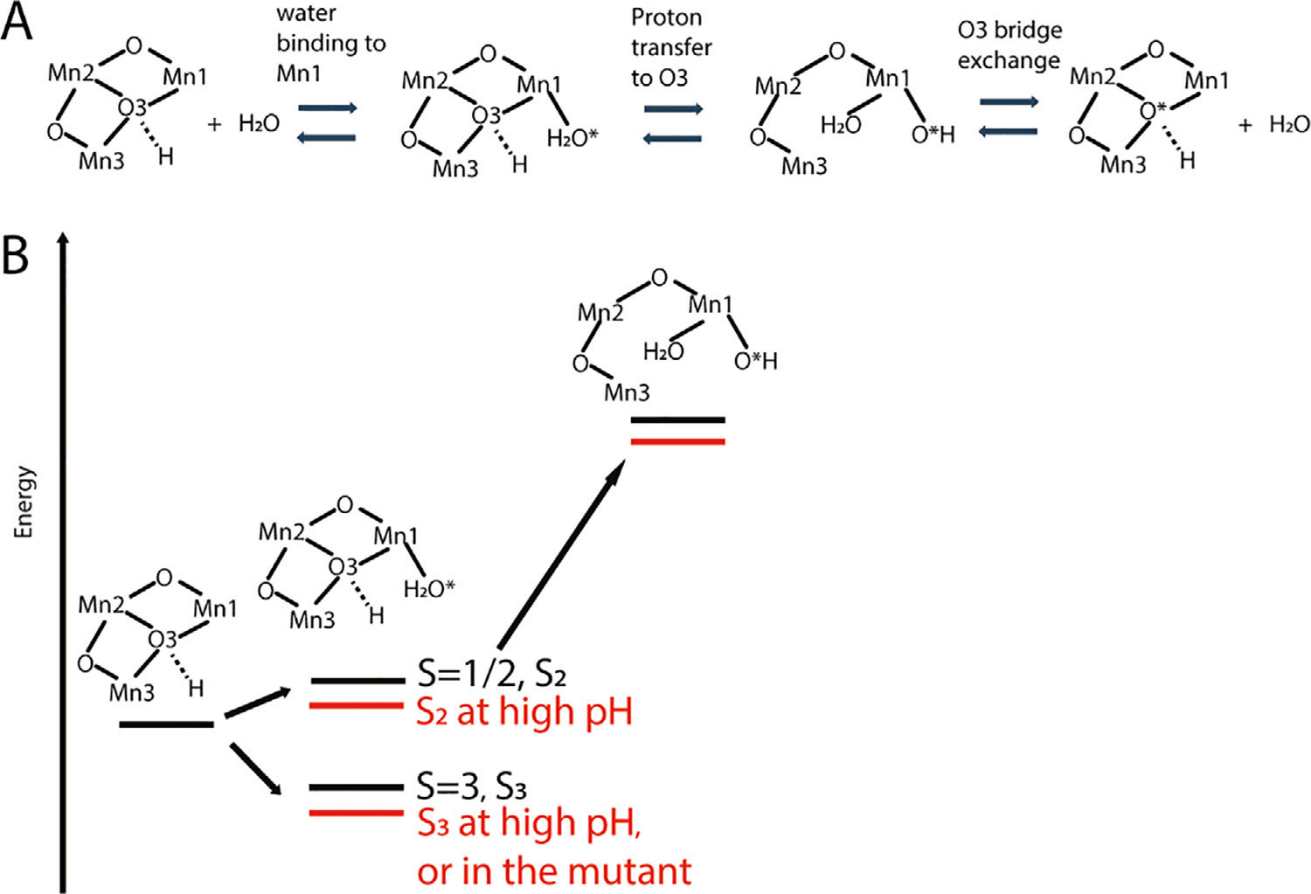
(A) Mechanism of substrate exchange in the OEC. O3 is assigned as a slow exchanging substrate oxygen, Ws. Steps needed to exchange this oxygen are shown. Note that 6‐th ligand on Mn1 is present as a ground state in the S_3_‐state of the Kok cycle. (B) Energy diagram showing how water ligand on Mn1 is over stabilized in the S_3_ state, affecting the population of this state. Changes in the pH and mutations in amino acids in the vicinity of the OEC can affect the substrate exchange rates by changing the energy level of states involved in substrate exchange. The energy difference associated with proton transfer to the O3 bridge (H_2_O state, large black arrow to the left) for two models of the S_3_ state with protonated His337 was found to be ∼14 kcal/mole, see Supporting Information for energy values and coordinates of these models.

The advantage O3 has over O5 as a potential slow‐exchanging substrate oxygen is that it is the only bridging oxygen in the OEC which can be reliably shown to be protonated—a necessary pre‐requisite for exchange. Protonation of the His337 at Nδ results in movement of the proton from Nε to O3 (Table S2). We foresee the exchange to have same start as in the earlier proposed scheme for O5 exchange [21]—with the coordination of water to five‐coordinate Mn1. Then, instead of proton transfer to O5 and a complex open‐to‐closed cubane interconversion needed for the O5 exchange, is it simply enough to transfer a proton from water coordinated to Mn1 to protonated O3. The water ligand in the O3 position is ready to dissociate, while the remaining ─OH coordinated to Mn1 can occupy its site, enabling a sub‐second exchange rate, Figure 2A.

Let us see if assignment of the O3 as Ws can satisfy, at least qualitatively, all available observations: i) Ws exchanges the fastest in the S_0_ state. This state has a high content of Mn^III^, and thus the highest exchange being detected in this state is not surprising. ii) Increasing the content of Mn^IV^ in the S_0_ to S_1_ oxidation step slows down the exchange while binding of the water to Mn1 is inefficient in the S_1_ state. Thus, the S_1_ state shows the slowest rate of the O3 exchange. iii) With the transition into the S_2_ state, the content of the Mn^IV^ further increases, but so too does the propensity of this state to bind H_2_O at Mn1. Higher population of Mn1‐H_2_O state allows for a counterintuitive effect of faster substrate exchange despite the increased Mn oxidation state. This was already shown in the earlier calculations where O5 exchange was modeled [21], iv) Ca^2+^ for Sr^2+^ exchange opens the OEC due to coordination of the larger Sr^2+^ ion, which supports longer Sr─O distances and increases the population of the Mn1‐H_2_O state, thus resulting in faster O3 exchange rates.

The overall process can be envisioned as a two‐state equilibrium where O3‐Mn1‐H_2_O* can either keep exchanging Mn1‐H_2_O* with other waters (fast exchange) or exchange it with O3 (slow exchange).

(H^+^)O3‐Mn1 + H_2_O* = (H^+^)O3‐Mn1‐H_2_O*

(H^+^)O3‐Mn1‐H_2_O* = H_2_O3‐Mn1‐O*H = O3*‐Mn1 + H_2_O

* denotes incoming water

Such an exchange mechanism involving O3 as a slow exchange water requires fewer steps than the one proposed earlier for O5, and it also does not require the formation of a “closed” cubane state, which so far has not been observed experimentally. Below we analyze if available mutagenesis studies are consistent with O3 assessment as a critical oxygen center in the OEC.

### 2.3 Data From Available Mutagenesis Studies Do Not Contradict Assignment of the O3 as Ws

Mutants of His337 that have been reported: H337R, H337Q, H337N, H337E, H337D, H337Y, H337V, and H337L, plus the H337F mutant originated by pseudoreversion [34], Only the H337R (arginine, Arg), H337F (phenylalanine, Phe), H337Q (glutamine, Gln), and H337N (asparagine, Asp) mutants could be propagated in the absence of glucose. These have shown diminished rates of O_2_ evolution from ∼50%–20% of the wild type [34], Both Arg and Gln are good hydrogen bond donors that can potentially substitute for His337, while Phe was proposed to cause the re‐structuring of the OEC coordination with a different amino‐acid fragment or water taking the role of the H‐bond donor [34], Thus, an H‐bond to O3 seems to be essential for OEC oxygen evolving function based on the H337 mutagenesis studies.

The D1‐V185 residue has been the focus of several recent experimental and theoretical studies as its mutations change the rate of O_2_ evolution and substrate exchange in an intricate way [35, 36, 37, 38]. The V185 residue is located adjacent to the cluster and may also regulate the access to the open coordination site at Mn1. In PSII from *Synechocystis* sp. *PCC 6803*, mutation of the D1‐V185 to the polar residue asparagine slows down the release of O_2_ from a halftime of 1.2 ms to about 27 ms. On the other hand, mutation of V185 to threonine resulted in only a modest effect on the oxygen release (from 1.2 to 1.5 ms) [36]. One theoretical study found that by rotating the V185 residue away from the position determined by crystallography, the insertion of water from calcium onto Mn1 becomes easier and occurs in the sub‐microsecond timescale rather than the millisecond timescale [23]. Substrate exchange data for the D1‐V185N mutant strain of *Synechocystis* sp. *PCC 6803* have shown that Ws exchange was faster by a factor of ∼4.4 for the S_1_ state and ∼2 for the S_2_ state [35]. This was explained by possible stabilization of the Mn1‐H_2_O(or ‐OH) state by the proximal asparagine. However, in the S_3_ state, the exchange of both slow and fast exchanging substrate oxygens was twice as slow. This apparent contradiction can be rationalized by overstabilization of the Mn1‐H_2_O(or ─OH) state in the S_3_ which makes both exchanges with water and O3 slower, Figure 2B. Overstabilization of Mn1‐OH should also make O─O bond formation harder in agreement with slower O_2_ evolution rate in this mutant. So, results of substrate exchange in D1‐V185N mutant do not contradict the substrate exchange mechanism proposed in Figure 2A.

pH dependence of substrate exchange was also analyzed with two pH points of pH = 6.0 and pH = 8.6 being compared in Ca‐PSII and Sr‐PSII core preparations from *Thermosynechococcus elongatus* [20]. In the S_2_ state, increasing pH slows fast exchanging substrate slightly and speeds up the exchange of W_S_. This is consistent with stabilization of the Mn1‐H_2_O(or ─OH) state by high pH, Figure 2B. This was noted to be consistent with conversion of the S_2_ state to high spin (HS)‐S_2_ [20], and consistent with original proposal of the HS‐S_2_ as a Mn1‐H_2_O(or ─OH) state [24]. In the S_3_ state, similar to the case of the D1‐V185N mutant discussed above, the high pH decreases both W_f_ and W_S_ exchange rates. Overstabilization of the Mn1‐H_2_O(or ‐OH) state in the S_3_ by high pH might be responsible for slower exchange of both O6 and O3 substrate oxygens with outside (unbound) water, Figure 2B. High pH = 8.6 data might also indicate that electron back donation to Tyr_Z_ might not be essential for a substrate exchange in the S_3_ state. The S_2_Tyr_Z_^•+^ state is more stabilized at basic pH as was confirmed by EPR data [39], while substrate exchange is slowed in the S_3_ state by basic pH.

### 2.4 Energetics of the Substrate Exchange at O3

In general, in order to exchange water bound to a Mn center, it should be bound as a terminal and fully protonated ligand on Mn(II) or Mn(III), as Mn(IV) is generally considered to be exchange inert. Additionally, one has to consider that water exchange can occur via two different mechanisms, namely through an associative mechanism, where a new water binds first before the original water ligand leaves, or a dissociative mechanism, where water is released first before another water binds. As O3 is a ligand to five‐coordinate Mn1, the associative mechanism is a straight forward one. In fact, all previously analyzed schemes of O5 exchange started with water binding to Mn1 [21, 40]. We computed several additional states for the S_3_ level of the OEC oxidation and spin state *S* = 3 which is most consistent with experiments [41], Figure 1B; Table 1. For more S_3_ state models presented by our group, see citations [25, 42]. Calculations were done for models with protonated His337, featuring a water ligand bound to Mn1 versus the Mn1‐OH state with a proton transferred to O3 (see Figure 2A for the overall exchange proposal). The energy difference associated with proton transfer to the O3 bridge, forming H_2_O ligand in this position, was found to be ∼14 kcal/mole, in agreement with characteristics of slow water exchange kinetics. As it was shown earlier that the O5 oxo‐bridge protonation step is the highest energy state in the model of the substrate water exchange computed previously at ∼18‐20 kcal/mol [21], the model of the O3 exchange represents a viable option.

**TABLE 1:** Energies of selected intermediates demonstrating the rearrangement of protons for modeling of the substrate exchange and rearrangement of oxygens for modeling the O─O bond formation. All energies are in Hartree unless energy units are indicated.

S_3_‐state model with Mn4‐H_2_O, ─OH, S = 3, charge 2+ and protonated His337 shows proton transfer from Mn1‐H_2_O to form Mn1‐OH;O3H			
Initial, E −8387.0807	Final, E −8387.0583	ΔE +0.0224	ΔE +14 kcal/mol
S_3_‐state model with Mn4‐H_2_O, ─OH, charge −1 and His337‐Nδ‐H^*^ configuration shows preferential O3‐O6 peroxide formation			
Initial, E, S = 3 −8120.085278	Final, E, S = 1 −8120.08625	ΔE −0.00098	ΔE −0.02 eV
	Final, E, S = 1 −8120.09402	−0.00874	−0.24 eV

* Lack of the H‐bond between the His337‐Nε‐H and O3 makes His337 drift away from our unrestrained cluster OEC model. Thus, His337 was removed from the model for this set of calculations.

### 2.5 Mechanism of O─O Bond Formation With O3 as a Substrate Oxygen

**FIGURE 3:**
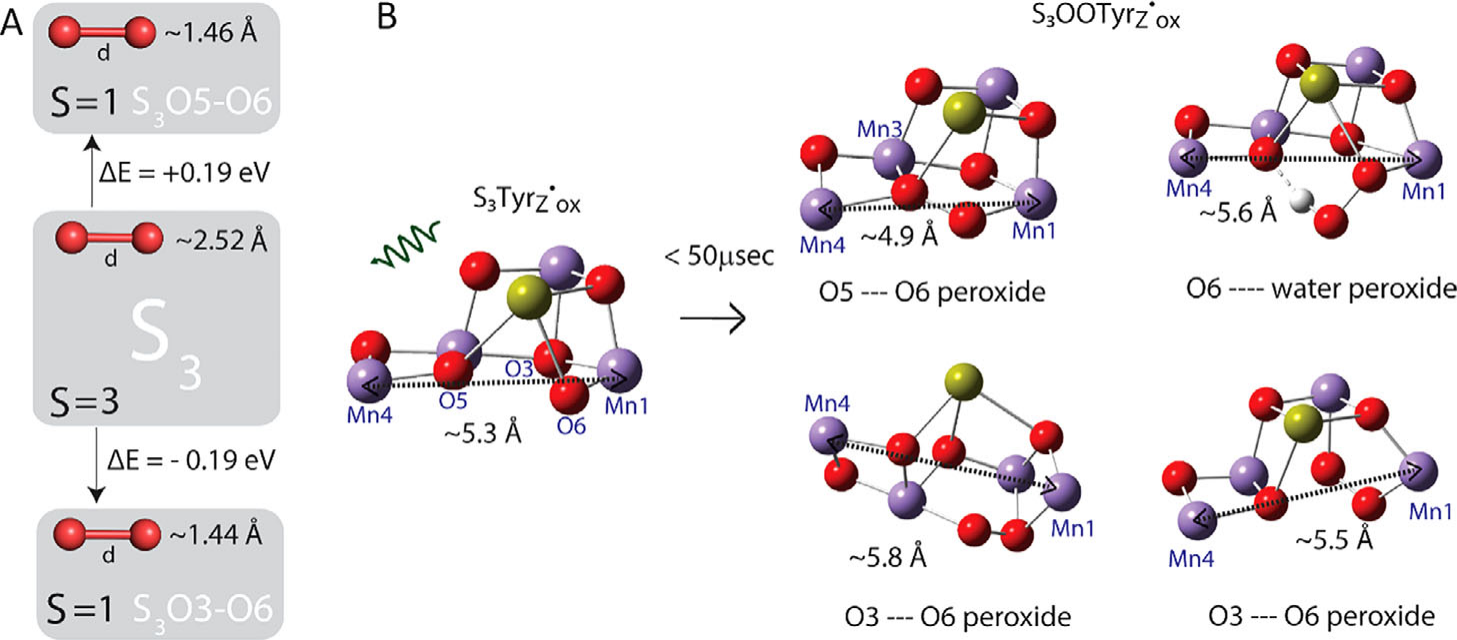
(A) Scheme comparing the energies of the S_3_‐state model with energies of the corresponding peroxides computed using BP86. O3–O6 peroxide formation lowers the energy when O3 is not H‐bonded to His337. (B) shows S_3_‐state DFT model featuring the Mn1═O fragment at early time in the S_3_ to S_0_ transition after ∼ns photoinduced dynamic results in Tyr_Z_ oxidation. Right side shows different DFT optimized peroxide models with indicated Mn1‐Mn4 distances. All ligands to Mn and Ca except bridging oxygens are removed for clarity. Top line peroxides are expected to be formed at higher energy while the lower ones (O3–O6) are formed with negative ΔE. O3–O6 peroxides satisfy TR‐XRD structural trend[4] on elongated Mn1─Mn4 distance in the S_3_ to S_0_ transition.

Formation of the O─O bond between the O5 and O6 (or O*x*) has been extensively studied by multiple research groups, Figure 3 [11, 43, 44, 45, 46]. It was also used by our group to explain the time‐resolved x‐ray emission data for the S_3_ to S_0_ transition [25, 47, 48, 49]. Some PSII TR‐XRD data placed these two oxygens in the 1.4–2.2 Å range, indicating very close contact even in the S_3_ state [5, 50, 51]. Thus, this mechanism of the O─O bond formation seemed to be reasonable, taking aside the contradictions of the O5 not meeting the properties of the slow exchanging substrate water discussed above. New TR‐XRD data [4] also tainted the proposal of the O5‐O6 coupling for O_2_ formation by detecting extension in the Mn1─Mn4 distance of the OEC cluster at all time points in the S_3_ to S_0_ transition instead of predicted contraction [49] if O5–O6 peroxide would be an intermediate, Figure 3. This contradiction of the O5‐O6 peroxide structure and TR‐XRD of the S_3_ to S_0_ transition was noted, and a different, protonated form of peroxo species forming between the O6 and incoming water via water nucleophilic attack mechanism was presented [49], Figure 3B. However, no energetic analysis was provided, and such a state, while in general plausible, does not meet the current understanding of the substrate exchange in the PSII.

Some studies based on the re‐analysis of the electron densities in XRD questioned the existence of the O6 oxygen in the S_3_ state [14, 52, 53, 54, 55, 56] but were followed by additional re‐analysis confirming the O6 [57]. Two studies also pointed to potential partial occupancies of the O1, O2, and O3 oxygen sites in the OEC structure (though deviations are ∼15%) and light dependent changes in the occupancy of some bridging oxygens [55, 56]. From these observations a mechanism of “expansion ‐contraction cycle” in low oxidation state paradigm was proposed and this mechanism currently awaits the detailed DFT validation of the energetics for the O─O bond formation and other relevant Kok cycle transitions. Incidentally this mechanism also considers O3 as a substrate site [55, 56].

Our group hypothesized O6 in the S_3_ state even before the first S_3_ XRF results to explain the evolution the OEC electronic structure measured by the TR‐XES [48]. It was also hypothesized in all theoretical studies on the O─O bond formation by the Siegbahn group [23]. Modeling of the Mn XANES [58] and EXAFS and Sr EXAFS data obtained in the S_3_‐state in both spinach and cyanobacterial PSII samples favor OEC structures with O6 in the cluster [42]. Thus, taking all the available data into account, experimental and theoretical evidences for the Mn1 coordination sphere expansion and O6 recruitment in the S_3_ state remain strong.

O3, similarly to O5, is located in very close proximity to O6, with O5—O6 vs O3—O6 distances differing at ∼0.1–0.3 Å depending on the OEC DFT model of the S_3_ state. While O5─O6 coupling into the peroxide takes place on the outside of the open cubane, O3─O6 coupling will take place almost inside the open cubane unit, assisted by all Mn1‐Mn3 and Ca^2+^ ions. Several geometries of O3‐O6 peroxides models were computed (Figure 3, Table 1) and some are found lower in energy in comparison to the S_3_‐state model, highlighting a smaller energy gap than reported by us before for O5─O6 peroxides [25]. A scan of the energy change along the O3─O6 bond distance found that ∼1.7–1.8 Å range of O─O distance has high energy with a maximum energy of ∼11.8 kcal/mol, Figure S1. This energy is comparable to earlier reports for O5─O6 [33, 43, 59]. The small energy gap between the S_3_ state and its peroxo isoform is significant to facilitate the O─O bond formation in the S_3_Tyr_Z_^•+^ state during the S_3_ to S_0_ transition with later transfer of the electron from the S_3_OO state to the oxidized Tyr_Z_^•+^ [25, 47, 49].

O3–O6 peroxides also fit better with the most recent TR‐XRD results showing the elongation of the Mn1─Mn4 distance in the early time points of ∼50–250 µsec [4], while O5─O6 peroxide should result in shortening of this distance. One more mystery of the TR‐XRD results is that second partner oxygen is not visible anywhere in the data but O6 electron density starts decreasing as early as ∼250 µsec (when compared to S_3_ state) and is clearly decreased at 500 µsec [4]. This can be the case if in reality a second water (O6,2) also binds to Mn1 and pushes the original O6,1 exactly at the time of the O─O bond formation toward the O_2_ out, taking the O3 position after O_2_ is formed. As this process would lead to minimal displacement of O3, the original O3 and new O6,2 taking its place would not be distinguishable in TR‐XRD, masking the density dynamics of the second substrate oxygen.

After the release of the oxygen, the replenishment of the O3 position (which is bridging between three Mn ions) is overall not different from the prior proposals as to the replenishing of O5 (bridging between two Mn ions). When O_2_ is released, the water or OH─ ion will occupy the O3 empty site. Water might come directly from the cluster environment, which is enriched in water (including structurally resolvable H_2_O), or it might come from first been ligated to Mn1 in the later stages of the S_3_ to S_0_ transition in association of “pushing” the peroxide/O_2_ group out of the OEC toward His337. Such water will likely spontaneously deprotonate to accept coordination with three Mn ions. Note all this is analogous to the proposed O5 site replenishment: directly from environment or via coordination to Mn4 center first and then assuming bridging position.

### 2.6 Analogy of the His337 and the Distal His in Myoglobin and Peroxidases

Assignment of the O3 as a slow W_S_ makes it the site of the oxygen production in the PSII. Intriguingly, myoglobin, another protein that handles O_2_, has a His site for H‐bonding with O_2_. Thus, when O_2_ is produced in the PSII, His337 can regulate its reactivity and assist its exit via H‐bond. The distal histidine (His‐E7) in myoglobin plays crucial roles in stabilizing bound oxygen, acting as a gate for its binding, and preventing harmful interactions. The distal E7 histidine in vertebrate myoglobins and hemoglobin has been strongly conserved during evolution and is thought to be important in fine‐tuning the O_2_ affinities of these proteins. A hydrogen bond between the Nε proton of the distal histidine and the second oxygen atom may stabilize O_2_ bound to the heme iron [60]. The distal histidine, which is not unique to myoglobin, is conserved in nearly all globins and in all heme peroxidases, appearing to have nearly opposite structural roles in these classes of proteins. In peroxidases, the distal His has the role of activating bound H_2_O_2_ for heterolytic bond cleavage. Myoglobin also has some residual peroxidize activity. Prevention of autoxidation would mean prevention of proton shuttling, since protonation of bound O_2_ is the first step in autoxidation of globins. Note that all His337 mutants have shown lower stability under illumination [34] hinting at potential lack of stabilization mechanism for safe handling of produced O_2_.

## 3 Summary

In the OEC structure, the bridging O3 is a frustrated center placed in between four dissimilar positive charges: three Mn ions and one proton. Its state is highly modulated from been protonated (estimated pKa∼6); been H‐bonded with His337 and lacking stabilization of the H‐bond, Figure 1B. Environment of the His337 Nδ nitrogen is involved with extended H‐bond network (Figure 1C) and thus can be modulated in the Kok cycle to affect, via Nδ—Nε tautomeric states, the charge on the O3. We show that when O3 lacks the stabilization of the H‐bond to His337, the energy of O3‐O6 peroxide formation is negative even in the S_3_ state. In the PSII OEC the mechanism of O─O bond formation between the O3 and O6 activated centers is currently the one which is most consistent with all available experimental data, including the data on substrate exchange, EPR and TR‐XRD. The mechanism highlights how the protein environment can steer the O─O bond formation by charge control on the O3 via H‐bond and open coordination of Mn1 via 5–6 coordinate conversion dynamics.

## Conflicts of Interest

The author declares no conflicts of interest

## Supporting information




**Supporting File 1**: anie73276‐sup‐0001‐SuppMat.pdf.

## Data Availability

The data that support the findings of this study are available from the corresponding author upon reasonable request.
