# Supplementary material for: New Water Oxidation Mechanism in Photosystem II Resolves Major Experimental Controversies
Source: Angew Chem Int Ed Engl. 2026 Jun 22;65(35):e1930324. doi: 10.1002/anie.1930324 (PMC13502591; doi:10.1002/anie.1930324)
Supplement: Supplementary file 1 [file ANIE-65-e1930324-s001.pdf]

Supporting Information for

New water oxidation mechanism in Photosystem II resolves major experimental controversies.

Yulia Pushkar

[1] Department of Physics and Astronomy, Purdue University, West Lafayette, Indiana 47907, USA

*Correspondence to: ypushkar@purdue.edu

Materials and methods

For analysis of energies and geometries of PS II intermediates, DFT calculations of molecular models were done with Gaussian09 unrestricted BP86 Becke's 1988 functional[1] with the gradient corrections of Perdew[2] and the def2tzvp basis set[3] used for all atoms, **Tables 1, S1, S2.** The previously discussed models of the low spin (LS) S_2 state (S=1/2) and S_3-state (S=3) were used for some comparisons.[4-6] The size of our model is maximized to the level when large basis set calculations can be carried out over a practical time period. In the past we documented smaller models results in Davis et al.[7] to be very consistent with later enlarged models[8-10] yielding essentially the same spin configurations, Mn-Mn bond geometries and energetics. At the level of O-O bond formation / spin configuration analysis, much smaller models have proven to be very insightful, see earlier works of Siegbahn[11].

Table S1. Representative ^{17}O hfs constants in MHz from DFT calculations with UBP86 potential and def2tzvp basis set for all atoms of the **S_2 state models (S=1/2)**.

Oxygen center numbering 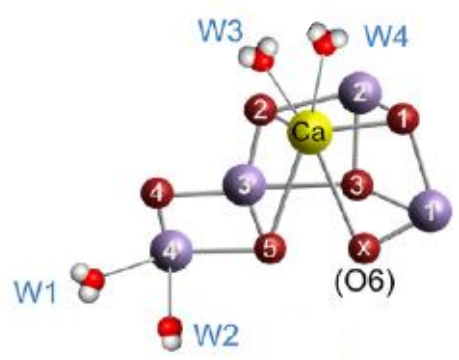	^{17}O a_{iso}	A_{xx}	A_{yy}	A_{zz}
S_2 state (Mn4-OH,-OH, His337 unprotonated)				
O5	-5.5	54.4	-21.1	-33.2
O4	12.7	66.4	-14.2	-52.2
O2	-6.1	28.3	-8.3	-20.0
O1	-15.3	32.7	11.3	-44.0
O3	-7.9	21.1	1.0	-22.2
Mn4-OH	17.3	34.2	-9.5	-24.8
Mn4-OH	20.6	23.9	-8.1	-15.8
S_2 state (Mn4-OH,-H_2O, His337 unprotonated)				
Mn4-H_2O	-4.9	1.1	0.3	-1.46
S_2-state [Mn4-OH,-OH, His337H^+]				
O5	-3.5	16.0	13.1	-29.1
O4	10.0	20.6	17.7	-40.3
O2	16.2	48.7	-2.3	-46.4
O1	-1.9	33.9	-15.4	-18.4
O3	-6.4	10.2	0.8	-11.0
Mn4-OH	-23.6	33.2	12.1	-45.4
Mn4-OH	-19.8	20.8	6.3	-27.1

Table S2. Mn-Mn and other relevant distances, Å

	S=1/2, S_2 Mn4-OH,-OH, His337	S=1/2, S_2 Mn4-OH,-OH, His337H^+	S_3 Mn4-H_2O,-OH, Mn1=O, His337	S_3 Mn4-H_2O,-OH, Mn1=O, His337H+
Mn4-Mn3	2.75	2.74	2.71	2.70
Mn3-Mn2	2.78	2.87	2.78	2.87
Mn2-Mn1	2.73	2.83	2.82	2.89
Mn1-Mn3	3.33	3.53	3.54	3.74
O3-H	1.88	1.05	1.87	1.03
O3-Nε	2.92	2.63	2.91	2.66

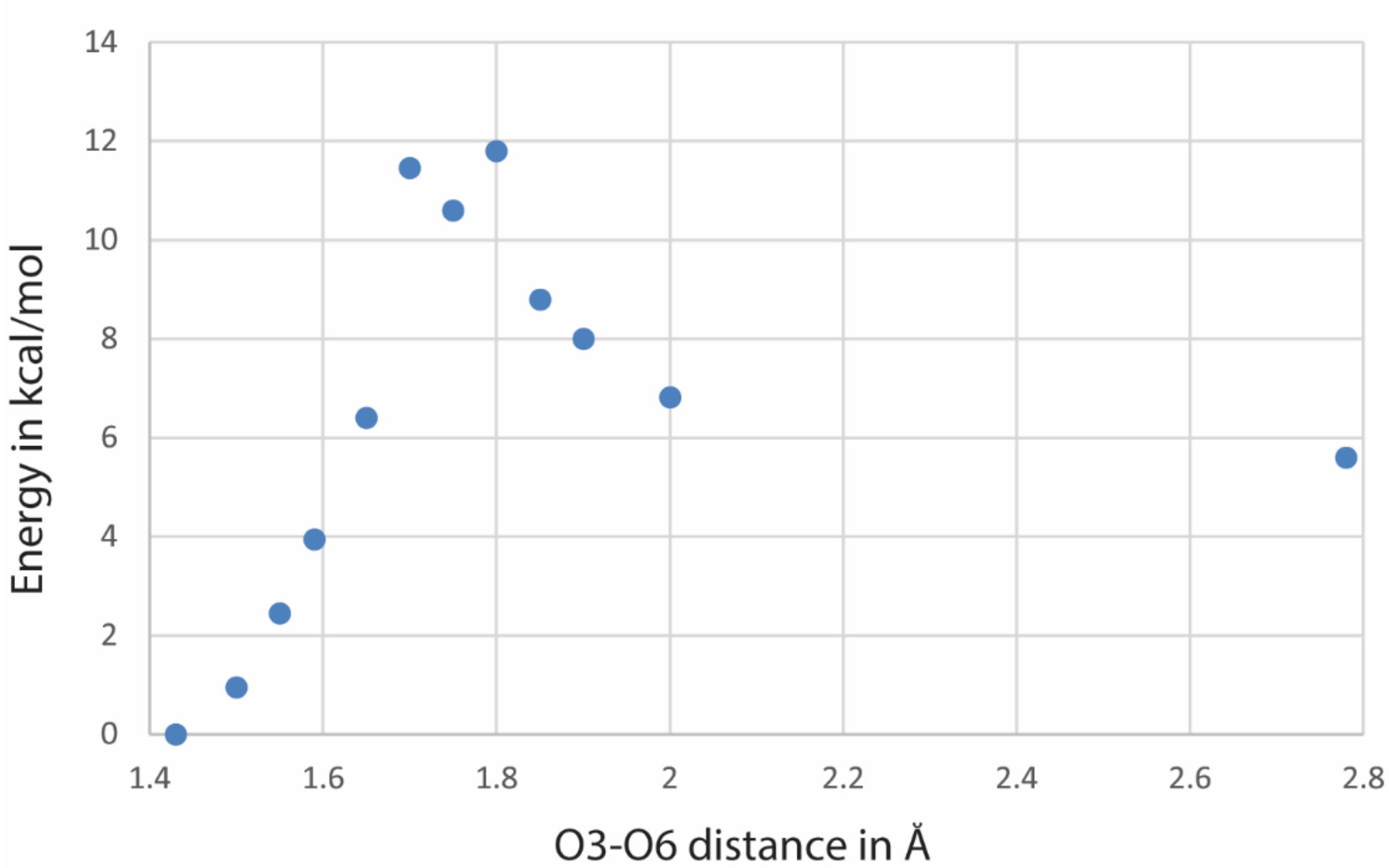

Figure S1. O3-O6 distance scan between the S_3OO and S_3 models. S_3-state model with Mn4-H_2O,-OH, charge -1 and His337-Nδ-H* configuration shows preferential O3-O6 peroxide formation, **Table 1**. Scan was computed with S=1. Attempt to propagate the O-O bond formation with S=3 resulted in higher energies. Transition state energy was estimated at ~12 kcal/mol, coordinates of highest energy state (O3-O6 distance ~1.8Å) are deposited below.

Coordinates deposition:

S_2-state model [Mn4-OH,-OH, His337H$^+$], S=1/2, charge 0.

Mn	-1.15662500	-3.09941500	-1.00554800
Mn	-0.50551400	-0.46816500	-1.42507600
Mn	-1.24773200	2.11990400	-0.41724000
Mn	0.18307100	0.95834800	1.73501200
Ca	-2.94413000	-0.41400500	0.92947000
O	-0.89845200	-1.46603000	0.02910800
O	-1.14737600	-1.79987200	-2.35497400
O	-1.95911100	0.64010300	-1.12900800
O	-1.46773000	1.54445500	1.31437000
O	0.41785200	1.04980200	-0.23757300
H	-4.66689000	-4.36980100	-3.35622700
C	-5.29701600	-4.51107900	-2.46599200
H	-6.28150600	-4.86953800	-2.80361600
C	-5.44293000	-3.20980500	-1.67977400
C	-4.15238000	-2.58469400	-1.14406600
O	-4.20552500	-1.42532100	-0.68679800
O	-3.11003500	-3.34376700	-1.19009000
C	-0.24608400	0.62013100	6.40302900
C	-1.54438600	0.52315700	5.60067200
C	-1.38751900	0.29822900	4.09872000
O	-2.32707200	-0.20537400	3.44779700
O	-0.25145200	0.67177500	3.59324900
H	5.93035300	-2.93656500	3.06951500
C	5.37844300	-3.38763100	2.23295000
C	5.81236600	-2.65352600	0.96827700

C	3.84825800	-3.28647600	2.47282400
C	3.24871400	-1.91815900	2.30419600
N	3.96569000	-0.73374000	2.42909300
C	1.94894300	-1.53341700	2.05701400
C	3.11833400	0.30398100	2.23520200
N	1.88680000	-0.15083900	2.02120300
N	5.61335500	-3.31373600	-0.20513400
C	5.55701600	-2.61584600	-1.49321600
H	6.35333700	-1.85968000	-1.48744600
C	4.18914600	-1.94704800	-1.72643200
C	3.03902600	-2.95782100	-1.79545900
C	1.63725900	-2.43041100	-1.48975700
O	1.38861000	-1.19780100	-1.68492800
O	0.83058800	-3.31286400	-1.07425100
C	5.68766700	4.02952300	-2.01344100
C	4.50419300	3.13100600	-1.88688000
C	3.54310200	2.99280100	-0.90898700
C	3.03040300	1.53985900	-2.43540200
N	2.64066700	2.00695500	-1.25981200
H	0.93858900	7.35968900	1.11761400
C	0.61011100	6.39160400	0.71086800
H	-0.47776500	6.42565700	0.56809500
C	1.00416600	5.25613100	1.65366800
C	0.57018200	3.85904600	1.21693700
O	-0.26492800	3.77853900	0.26195900
O	1.07472000	2.87623800	1.85907400
C	-4.01160300	2.77707700	-0.01045700

O	-4.25413000	1.70759000	0.57929700
O	-2.85524900	3.13157800	-0.49446600
C	-1.38466800	3.46767900	-4.95399600
C	-0.19492800	2.58756800	-4.55281300
C	-0.34553200	1.95373000	-3.17388100
O	-0.73614400	2.73446400	-2.23775900
O	-0.04344800	0.72751900	-3.05270300
O	6.25309300	-1.49456800	1.01647600
H	-4.83943900	-5.29940200	-1.85262200
H	5.20946000	-4.24444600	-0.15484300
H	1.31224400	1.46602400	-0.60508600
H	-5.93260800	-2.42863400	-2.28212100
H	-6.09210300	-3.35400600	-0.79934600
H	5.74679700	4.68812600	-1.13762300
H	6.63180800	3.46398200	-2.07055600
H	3.44647500	3.54554600	0.02034400
H	2.52783000	0.75295600	-2.99082900
H	3.40873300	1.34880400	2.24455800
H	1.06773700	-2.14470400	1.87713100
H	4.98892900	-0.68677000	2.34800500
H	5.78586700	-3.34703100	-2.28357500
C	-5.11224600	3.79382000	-0.25243700
H	-4.70981000	4.81232600	-0.30810100
H	-5.58797000	3.55981500	-1.21745600
H	-5.87504800	3.71682100	0.53177900
H	-2.30819300	2.87443800	-5.01783500
H	-1.54523800	4.26588600	-4.21773800

H	-0.02883200	1.77763900	-5.27636200
H	0.72456300	3.19859100	-4.52262900
H	0.33640200	-0.30977000	6.33039200
H	-2.19857000	-0.27584800	5.98039300
H	0.38652100	1.43908600	6.03638100
H	-2.12337400	1.45880600	5.70129600
H	3.64732500	-3.65857100	3.49215400
H	5.65650700	-4.45258500	2.19560200
H	3.31077700	-3.96864200	1.79669500
H	1.06526500	6.26310400	-0.28131400
H	0.56080000	5.40616200	2.65366700
H	2.09235600	5.22539700	1.82443100
H	4.01091200	-1.22447300	-0.91586600
H	3.19539500	-3.79381300	-1.09779100
H	2.97785800	-3.41689800	-2.79746500
H	4.23966500	-1.35968600	-2.65620700
O	-2.96302400	-2.63266000	1.95547900
H	-2.68944800	-2.54391700	2.88351900
O	-1.10781600	-4.61675600	-2.05365400
H	-2.28967200	-3.25896400	1.51035200
O	-1.18227400	-4.03902000	0.65278000
H	-5.12102200	0.77869100	2.11836000
O	-4.94640200	-0.10753200	2.49931200
H	-4.35309000	0.03802100	3.26484900
H	-2.01876500	-4.72383700	-2.39010600
H	-1.38605900	-4.96091900	0.40682800
H	-0.46525900	0.80005800	7.46593800

H	5.62545300	4.66975600	-2.90826100
H	-1.20721900	3.92719300	-5.93744300
N	4.15377300	2.19283800	-2.84897400
H	4.64474600	2.02829400	-3.72193900

S_3-state model [Mn4-OH2,-OH, Mn1=O, His337H$^+$], S=3, charge 0.

C	-5.99325300	-2.40231500	-0.69733000
C	-4.59677100	-2.20161400	-0.10613300
O	-4.37629300	-1.19654400	0.59375900
O	-3.72686700	-3.11935700	-0.38892400
C	-1.11876300	3.77956200	4.57049500
C	-1.14133600	2.79778100	3.39647700
O	-2.14338700	2.10597200	3.16129000
O	-0.01937400	2.81029300	2.71880600
C	5.86314900	-2.06051200	1.47012000
O	6.28279400	-1.53198200	0.43069200
C	4.79396800	-0.95604200	3.57594700
C	3.93926200	0.03139400	2.84528400
C	2.59469200	0.02335500	2.54439500
C	3.35600100	1.93213600	1.82177800
N	2.25431100	1.20111200	1.91102000
N	5.20923400	-3.25325000	1.47496200
C	3.58597400	-3.69213900	-0.40275700
C	2.37102200	-3.95648100	0.48898300
C	1.06558500	-3.32424400	0.00862900
O	1.12368200	-2.21714500	-0.59795700
O	0.01168200	-3.97751900	0.32819700

C	4.94932500	0.88165700	-2.68406500
C	3.64279500	1.32367800	-2.77352700
C	3.73018300	0.09607300	-1.00068100
N	2.89578500	0.82744300	-1.72248400
C	1.54005900	5.15099100	-1.15881700
C	0.89306400	3.82058400	-0.77820600
O	0.03138200	3.34029900	-1.58753900
O	1.27124900	3.28705100	0.30704000
C	-3.76340700	2.59995000	-1.13171500
O	-3.99828600	1.94138300	-0.10465900
O	-2.64579800	2.60290500	-1.81151400
Ca	-2.54682300	0.32669700	1.48992100
O	-1.28432200	2.09577500	0.44206500
O	-1.91905800	0.07871900	-1.24786500
O	0.48355900	0.62277500	-0.60966400
O	-1.36374600	-2.65832800	-1.61747400
O	-1.31254100	-1.65109500	0.55991000
O	-0.24338300	0.25966700	2.01563000
Mn	0.31247300	1.65319800	1.24672100
Mn	-1.06222500	1.68163000	-1.29385300
Mn	-0.58572500	-1.14194100	-1.07639000
Mn	-1.80472800	-3.26851200	0.06517000
O	-2.23537900	-5.14744700	-0.99091800
O	-4.98961300	0.83159700	2.24785100
O	-2.29067700	-3.84973900	1.79498300
O	-2.71804500	-1.48046600	3.19592000
C	0.21537200	0.04028800	-5.11999000

C	-0.10931300	0.18504300	-3.63814400
O	-0.54080900	1.30273500	-3.22279200
O	0.09181800	-0.85687000	-2.92552600
H	-6.70111400	-2.33232400	0.14497400
H	-6.18403800	-1.50887900	-1.31448200
H	-0.25742400	3.50753300	5.20303400
H	-0.87754300	4.77359000	4.15924100
H	4.15676800	-1.82141400	3.81351900
H	5.10183000	-0.53968400	4.55165400
H	1.82230300	-0.71419600	2.74453500
H	3.41487200	2.91307600	1.36256900
H	3.50839700	-4.28531500	-1.32864300
H	3.59067900	-2.63844500	-0.71244700
H	2.19610800	-5.03099800	0.64519600
H	2.53435100	-3.51570100	1.48945400
H	4.90896500	-3.62771500	2.36966900
H	3.20386100	1.96721700	-3.53028100
H	1.36820000	0.76286700	-1.12976200
H	3.46948600	-0.44851200	-0.09874900
H	1.44672400	5.80567800	-0.27712400
H	2.62070500	4.95397800	-1.26580200
H	-0.39782300	-0.79894600	-5.48810200
H	1.25783100	-0.31316000	-5.18180100
H	-1.70427400	-4.92388900	-1.78374100
H	-3.14648300	-4.85953800	-1.22458400
H	-5.22938400	0.10461500	1.62738700
H	-5.02641300	1.62162600	1.66959200

H	-3.14883500	-4.31067000	1.76821300
H	-2.53566500	-2.35859600	2.74575300
H	-3.59922300	-1.56814500	3.59547000
C	6.14401800	1.11227700	-3.54769000
H	6.96617100	1.59539000	-2.99508400
H	6.53551100	0.17166300	-3.96784900
H	5.87555800	1.76659500	-4.38723500
C	-0.00294100	1.29561800	-5.96171400
H	0.62206900	2.12804500	-5.61035000
H	0.24657900	1.09742300	-7.01484600
H	-1.04769300	1.63072600	-5.91137700
C	-6.22519300	-3.67860600	-1.50367700
H	-6.06671800	-4.57788800	-0.89076900
H	-7.25745800	-3.71061000	-1.88300000
H	-5.54826600	-3.73232200	-2.36805900
C	-2.41266800	3.81158800	5.38062800
H	-2.64127300	2.82256500	5.80060000
H	-2.33027800	4.53135300	6.20922000
H	-3.26604700	4.10225200	4.75278200
C	-4.81024700	3.53750200	-1.71408100
H	-4.58129600	4.56406200	-1.38868100
H	-4.78215800	3.52235100	-2.81110800
H	-5.80743800	3.26540000	-1.34847200
C	0.97999300	5.82263400	-2.41108700
H	1.50486900	6.76993100	-2.60688000
H	1.08918400	5.17724500	-3.29295700
H	-0.09097500	6.03950400	-2.29834000

C	4.92648100	-4.03389200	0.26619400
H	4.96174100	-5.09775500	0.54904600
H	5.74940700	-3.84526200	-0.43761000
C	6.07918600	-1.41585800	2.84124500
H	6.75389900	-0.56540400	2.67133200
H	6.61827300	-2.12243000	3.49433300
N	4.39820800	1.26168200	2.38153500
H	5.35438800	1.59941900	2.42009000
N	4.97757400	0.10056300	-1.54011700
H	5.75871700	-0.41637300	-1.12746800

S_3-state model [Mn4-H_2O, -OH; Mn1-H_2O; His337H^+], S=3, charge 2+

C	-6.17923200	1.99915900	1.30659500
C	-4.81902800	1.79005200	0.65246000
O	-4.62705000	0.78166700	-0.05823600
O	-3.93497800	2.70974700	0.88658800
C	-1.16123400	-2.89052700	-4.97159600
C	-1.10260500	-2.02641600	-3.72346100
O	-2.10433000	-1.44735900	-3.26328400
O	0.10527300	-1.94395300	-3.20231800
C	5.39391700	2.83463400	-1.15477200
O	5.43888600	1.95430400	-0.28066400
C	4.66454700	2.09208500	-3.50403600
C	3.88477600	0.91264000	-3.01749000
C	2.63789600	0.83295900	-2.44317200
C	3.36914200	-1.21638800	-2.57255800
N	2.32972200	-0.48792500	-2.16744300

N	4.92183000	4.08443800	-0.90150500
C	3.22556800	3.92628500	0.96142000
C	2.00095900	4.24817500	0.10072000
C	0.78011900	3.38655100	0.38989300
O	0.95939000	2.19630900	0.79732900
O	-0.35233800	3.93031400	0.14026100
C	4.64770600	-1.82421600	2.85989400
C	3.33305700	-1.92370500	2.47184100
C	4.20186900	-0.22458300	1.33976500
N	3.08710700	-0.92887100	1.53925400
C	2.09092500	-4.97679900	-0.24178000
C	1.32595300	-3.67092700	-0.14223900
O	0.58880400	-3.44281400	0.85126900
O	1.49995700	-2.84752500	-1.12495600
C	-3.23004200	-3.20242800	1.03914800
O	-3.70846700	-2.44791400	0.17061600
O	-2.00639000	-3.12822400	1.50167300
Ca	-3.04934900	-0.58249600	-1.10015700
O	-1.04087600	-1.93502200	-0.80192200
O	-1.75171500	-0.46684800	1.33475300
O	0.65806600	-0.57726100	0.35206400
O	-1.49538200	2.26166200	1.92497200
O	-1.48166300	1.45935200	-0.32100500
Mn	0.48740500	-1.24348000	-1.46118900
Mn	-0.68122400	-1.86599300	1.02068300
Mn	-0.56178600	0.92678700	1.22997000
Mn	-2.09987200	3.02359200	0.35924300

O	-2.61300300	4.84150300	1.26382800
O	-5.21584000	-0.91085000	-2.18533300
O	-2.62700000	3.56190800	-1.36217400
O	-2.86848500	1.29553200	-2.73215800
C	0.50698700	-0.72174300	4.99977400
C	0.22035900	-0.67605100	3.51229100
O	-0.00936600	-1.76843900	2.89466700
O	0.23569300	0.47970500	2.96223000
H	-6.91008200	2.02841400	0.48049900
H	-6.39464100	1.06561300	1.85200100
H	-0.34871500	-2.55928300	-5.63849200
H	-0.87519700	-3.90946700	-4.65866100
H	3.95541800	2.92232600	-3.63765400
H	5.07634300	1.88310100	-4.50493500
H	1.93395500	1.63004200	-2.24163500
H	3.43652600	-2.29728800	-2.52874800
H	3.06956300	4.29307100	1.98830300
H	3.34504400	2.83821000	1.03108800
H	1.69990900	5.30324200	0.17386000
H	2.23524200	4.07265700	-0.96559700
H	5.01030600	4.78376200	-1.63316300
H	2.55465900	-2.61101200	2.78356000
H	2.15099500	-0.74708000	1.06054200
H	4.35876900	0.61992000	0.66613600
H	1.83371100	-5.40937800	-1.22297300
H	3.16156100	-4.71929300	-0.31401300
H	-0.23431100	-0.04904200	5.46340900

H	1.47646900	-0.21901000	5.15494700
H	-1.87549500	5.23313700	1.76948700
H	-3.33101300	4.62675300	1.89457000
H	-5.81143000	-0.51891000	-1.51700700
H	-5.61771000	-1.75570400	-2.45149300
H	-3.31349800	4.25444300	-1.35322100
H	-2.86815500	2.21976700	-2.31526300
H	-3.46639000	1.31253000	-3.49825500
C	5.46215700	-2.61009700	3.82786500
H	6.32752300	-3.08217600	3.33820100
H	5.84120500	-1.97557000	4.64349700
H	4.85218400	-3.40427200	4.27435600
C	0.47313000	-2.10633900	5.64204500
H	1.23565600	-2.77408400	5.21787900
H	0.66151000	-2.02120100	6.72090700
H	-0.50324400	-2.58813600	5.50272200
C	-6.33592800	3.21610400	2.21493000
H	-6.17316700	4.15464900	1.66634300
H	-7.35346200	3.24855100	2.62802600
H	-5.63583900	3.17791900	3.06154500
C	-2.51188500	-2.88761000	-5.68300300
H	-2.78937300	-1.87780600	-6.01448600
H	-2.46990400	-3.53659400	-6.56841800
H	-3.31022000	-3.25824200	-5.02649300
C	-4.04032200	-4.32943400	1.63472100
H	-3.76865800	-5.26619800	1.12357500
H	-3.81240600	-4.45644500	2.70000200

H	-5.10960800	-4.14476900	1.48388800
C	1.82811600	-5.96779600	0.88997600
H	2.40735200	-6.88563600	0.72094900
H	2.11732000	-5.55646500	1.86616500
H	0.76585800	-6.23853500	0.94466700
C	4.53473200	4.53903100	0.44122800
H	4.46558800	5.63536300	0.39829500
H	5.34579800	4.28614400	1.14143200
C	5.83336700	2.55109800	-2.58721700
H	6.60343100	1.76963100	-2.52888000
H	6.29936200	3.43778000	-3.04435100
N	4.32353400	-0.40387700	-3.08596000
H	5.20327000	-0.72031000	-3.48527500
N	5.15551300	-0.75301300	2.13000900
H	6.10896700	-0.40034000	2.17599700
H	-0.69485400	1.06195600	-1.41442300
O	-0.21298700	0.48752900	-2.15948700
H	-0.91581400	0.38637100	-2.84757900

S_3-state model [Mn4-H_2O,-OH; Mn1-OH; O3H; His337H^+], S=3, charge 2+.

C	-6.13367900	2.14735400	1.27653300
C	-4.77809500	1.90729200	0.62343700
O	-4.60689500	0.89183300	-0.08248000
O	-3.87534400	2.80974900	0.85324500
C	-1.54704500	-3.40856300	-4.15129000
C	-1.14105000	-2.00526700	-3.73428100
O	-1.88454700	-1.01905400	-3.89276800

O	0.06830000	-1.94514500	-3.21349400
C	5.45401700	2.73353600	-1.18838200
O	5.48096900	1.85660900	-0.31012300
C	4.70941200	1.99501800	-3.53413500
C	3.90563500	0.83413400	-3.04204600
C	2.65741900	0.78277000	-2.46739800
C	3.34645100	-1.28173900	-2.58707500
N	2.32222400	-0.53019500	-2.18544000
N	5.00769100	3.99394700	-0.94103900
C	3.30866200	3.87943900	0.92255800
C	2.09086000	4.22231900	0.06030700
C	0.85261600	3.38730300	0.35350900
O	1.00745200	2.19557200	0.76657100
O	-0.26846100	3.95300400	0.10127400
C	4.61262600	-1.88999900	2.84823000
C	3.29618700	-1.96432300	2.46060900
C	4.19960800	-0.28875500	1.32054500
N	3.07064200	-0.96906900	1.52332400
C	1.99149300	-5.00407400	-0.23859400
C	1.25348400	-3.68232500	-0.14524900
O	0.52123500	-3.43444200	0.84714600
O	1.44428400	-2.86731900	-1.13184100
C	-3.29186200	-3.11486000	1.03376400
O	-3.75476000	-2.35480000	0.16166100
O	-2.06691500	-3.06359600	1.49597200
Ca	-3.05758600	-0.50932500	-1.11789200
O	-1.07726900	-1.90135500	-0.81320400

O	-1.75769400	-0.40882400	1.31648600
O	0.64925000	-0.57330400	0.33440700
O	-1.44538900	2.31661000	1.89381300
O	-1.44828100	1.50358300	-0.34834700
Mn	0.46483700	-1.24444100	-1.47568300
Mn	-0.71615900	-1.83110900	1.00906600
Mn	-0.53942600	0.95959000	1.20515700
Mn	-2.03421900	3.08337600	0.32448200
O	-2.50988000	4.91568800	1.22044900
O	-5.23042800	-0.79827800	-2.20157400
O	-2.55030100	3.62425600	-1.39947700
O	-2.83832900	1.35686400	-2.75874400
C	0.49553900	-0.69269300	4.98274300
C	0.20981300	-0.64815800	3.49505200
O	-0.04231900	-1.73850800	2.88258900
O	0.24882400	0.50442800	2.93953600
H	-6.86382900	2.18769600	0.45028400
H	-6.36816400	1.22101200	1.82633700
H	-0.72130600	-3.82443700	-4.75117600
H	-1.56281900	-4.01509700	-3.22941600
H	4.01746000	2.83899400	-3.67169700
H	5.11676800	1.77290100	-4.53402200
H	1.96999600	1.59507300	-2.26965200
H	3.39164400	-2.36357500	-2.53815500
H	3.16028300	4.25419700	1.94769100
H	3.40579100	2.78948200	0.99737000
H	1.81152800	5.28367500	0.12845000

H	2.32142300	4.03699400	-1.00516100
H	5.11045100	4.68784300	-1.67599100
H	2.50387000	-2.63403200	2.77554800
H	2.13842600	-0.77037200	1.04372800
H	4.37376000	0.54915800	0.64293800
H	1.72539300	-5.43591600	-1.21774000
H	3.06718200	-4.76893600	-0.31200800
H	-0.23176900	-0.00273900	5.44317000
H	1.47514300	-0.20923100	5.13557000
H	-1.76445900	5.29449100	1.72427600
H	-3.23210500	4.71869900	1.85217600
H	-5.81780700	-0.39104600	-1.53512700
H	-5.64956700	-1.63595600	-2.46375200
H	-3.22244300	4.33076600	-1.39381900
H	-2.81900700	2.28285700	-2.34622100
H	-3.43580900	1.38250700	-3.52493200
C	5.41084200	-2.68784400	3.81993000
H	6.26630700	-3.17988900	3.33253100
H	5.80288300	-2.05738100	4.63256700
H	4.78473000	-3.46721700	4.27015000
C	0.43332000	-2.07325200	5.63154900
H	1.18195600	-2.75850000	5.21056800
H	0.62347700	-1.98690100	6.71000300
H	-0.55274400	-2.53556600	5.49447300
C	-6.26531200	3.37153700	2.17910200
H	-6.08336300	4.30394100	1.62609100
H	-7.28193900	3.42680800	2.59200700

H	-5.56609900	3.32299500	3.02591000
C	-2.87948900	-3.49041400	-4.89173400
H	-2.85967200	-2.90468500	-5.82077300
H	-3.09902800	-4.53471100	-5.15278800
H	-3.70444800	-3.11328900	-4.27291900
C	-4.12505800	-4.22217500	1.63463000
H	-3.87270700	-5.16672100	1.12792500
H	-3.89972800	-4.34880100	2.70050600
H	-5.19034000	-4.01632200	1.48289100
C	1.70847700	-5.98411100	0.89782400
H	2.26874700	-6.91443200	0.73315500
H	2.00612400	-5.57419300	1.87206800
H	0.64089100	-6.23273500	0.95376000
C	4.63009000	4.46272700	0.39951800
H	4.58345400	5.56003200	0.35140300
H	5.43584100	4.19656200	1.10093500
C	5.88746400	2.43427700	-2.61945700
H	6.64133400	1.63745800	-2.55740300
H	6.37152500	3.30904100	-3.08076100
N	4.31728200	-0.49141800	-3.10428000
H	5.19031300	-0.82771900	-3.50206600
N	5.14225900	-0.83290200	2.11330800
H	6.10275200	-0.49965400	2.15765900
H	1.05674500	0.36101500	0.18643300
O	-0.16871200	0.41545800	-2.14899500
H	-0.90473200	0.40729100	-2.86980400

S_3-state model with Mn4-H_2O, -OH, charge -1. S=3 and His337-Nδ-H* configuration

* Lack of the H-bond between the His337-Nε-H and O3 makes His337 drift away from our unrestrained cluster OEC model. Thus, His337 was removed from the model for this set of calculations.

C	5.11202800	-3.30343000	-0.50451700
C	3.68874500	-2.83088400	-0.82060300
O	3.54594500	-1.77003700	-1.46397100
O	2.73339100	-3.57786000	-0.38555500
C	0.53620400	4.26108300	-3.94863200
C	0.55760500	3.05386600	-2.99764200
O	1.43193000	2.17191800	-3.14584200
O	-0.37338900	3.10075300	-2.09968100
C	-6.77149700	-0.71428400	-0.20978100
O	-6.93611000	-0.07588300	0.83821400
C	-5.71394900	0.20288900	-2.37704200
C	-4.59034700	0.82439300	-1.59800000
C	-3.23601400	0.84204500	-1.84516000
C	-3.52670000	1.99391100	-0.02659800
N	-2.59384700	1.57108400	-0.86719900
N	-6.35442700	-2.01150200	-0.23163700
C	-4.36280300	-2.52842800	1.20863700
C	-3.47770000	-3.12340100	0.11156100
C	-1.98939900	-2.79791400	0.26690000
O	-1.67236100	-1.79703900	0.95624200
O	-1.20262500	-3.59100800	-0.36745300
C	-0.60416300	5.23147600	1.98504600
C	-0.27761800	3.82812700	1.46305600
O	0.71523100	3.23226800	1.99101000

O	-1.02200200	3.38029200	0.54251800
C	4.09792600	1.86167000	0.68016000
O	3.97435300	1.25348400	-0.40820800
O	3.20119700	1.99487800	1.59862600
Ca	1.95404700	0.20649400	-1.78969500
O	1.27533300	1.92811500	-0.31370600
O	1.87655100	-0.32730800	1.23317200
O	-0.38340400	0.70299800	0.97812400
O	0.77414900	-2.90631100	1.36588100
O	0.53881900	-1.56149000	-0.65688700
O	-0.40299000	0.47932900	-1.79263900
Mn	-0.51344600	1.71202300	-0.69872400
Mn	1.33655200	1.39269700	1.40223300
Mn	0.30177200	-1.13221600	1.12484000
Mn	0.74083800	-3.29296300	-0.43197900
O	0.97273600	-5.35520600	0.48791100
O	4.35632400	0.33699400	-2.93519700
O	0.77391000	-3.76528200	-2.28674100
O	1.41717000	-1.44288800	-3.60330300
C	0.31025600	-0.23321500	5.33222500
C	0.46548000	-0.04148200	3.82173900
O	1.07114400	0.99573500	3.41732000
O	-0.03298600	-0.96872900	3.10294000
H	5.66627900	-3.30294600	-1.45760200
H	5.56219800	-2.49762900	0.09861600
H	-0.51419000	4.46918700	-4.20586900
H	0.87428800	5.12879000	-3.35567800

H	-5.33608400	-0.73476400	-2.81607200
H	-5.98619900	0.84050000	-3.23750900
H	-2.65745700	0.37307600	-2.63496400
H	-3.33946200	2.57571500	0.86823900
H	-4.11524600	-2.99495600	2.17637600
H	-4.13657500	-1.45843100	1.31888600
H	-3.58545300	-4.21744500	0.04069000
H	-3.75694400	-2.71634600	-0.87707800
H	-6.15439800	-2.42448500	-1.13800200
H	-0.45617300	5.91587500	1.13242200
H	-1.68634800	5.25472200	2.19418300
H	0.93223100	-1.10576900	5.59627900
H	-0.73021200	-0.54724600	5.51174800
H	0.76860900	-4.83138600	1.30494400
H	1.95022800	-5.28547100	0.43399300
H	4.46145600	-0.54212200	-2.50036200
H	4.57648900	0.94561500	-2.19183100
H	1.55435200	-4.32574400	-2.44651400
H	1.11884000	-2.28861400	-3.13452200
H	2.24161700	-1.69373000	-4.05271600
C	0.68682800	0.97986800	6.18120000
H	0.05232300	1.84466100	5.94059400
H	0.57087000	0.75423500	7.25323500
H	1.72687100	1.28157800	5.99790300
C	5.24232400	-4.64827100	0.20869100
H	4.82020100	-5.46620800	-0.39389100
H	6.30060200	-4.88378100	0.40207400

H	4.71520800	-4.63543200	1.17282300
C	1.40001500	4.09262200	-5.19766600
H	1.04537700	3.25343700	-5.81383400
H	1.38001900	5.00571100	-5.81437400
H	2.44161600	3.87398900	-4.92615800
C	5.41911500	2.55998500	0.99417700
H	5.38292900	3.57607200	0.57043700
H	5.57316900	2.64640800	2.07712700
H	6.25439700	2.02371100	0.52517600
C	0.20684500	5.68558100	3.19770900
H	-0.07745200	6.70752700	3.49588500
H	0.04620300	5.01770300	4.05568700
H	1.28296700	5.67309800	2.97798900
C	-5.86751000	-2.70987900	0.96132600
H	-6.12511000	-3.77651300	0.85351800
H	-6.43653800	-2.30701100	1.81040300
C	-7.00950900	-0.06859800	-1.57622000
H	-7.53961900	0.87295500	-1.37566100
H	-7.68154600	-0.69465500	-2.18604900
N	-4.75749000	1.58048100	-0.44138300
H	-5.60332700	1.58907900	0.13003800

S_3-state model in the peroxo state with O3-O6 bond at ~1.44Å

with Mn4-H_2O, -OH, charge -1. S=1 and His337-Nδ-H* configuration

* Lack of the H-bond between the His337-Nε-H and O3 makes His337 drift away from our unrestrained cluster OEC model. Thus, His337 was removed from the model for this set of calculations.

C	7.00698500	-2.15054900	0.07852600
C	5.51216000	-1.98845300	0.38396800

O	4.85082900	-3.01633400	0.65026900
O	5.05902200	-0.77552500	0.37430500
C	-2.86922200	-0.77293400	4.99381200
C	-2.22251900	-0.75574800	3.60704600
O	-1.18230400	-1.39772700	3.38919200
O	-2.86331000	-0.01219600	2.74786000
C	-0.54044500	6.43084100	-0.18768000
O	-1.18747000	6.24133000	-1.23156900
C	-1.34321700	5.32360700	2.00187300
C	-2.09010600	4.17377900	1.38178100
C	-2.38664100	2.92985500	1.89372800
C	-3.04343400	2.90812700	-0.17587600
N	-2.96842500	2.14929000	0.91457300
N	0.81633300	6.46810300	-0.16675000
C	1.85923100	4.59752600	-1.47940300
C	2.67481400	3.95992400	-0.35145600
C	2.55362000	2.42975300	-0.24728300
O	1.54782700	1.91998000	-0.85032400
O	3.41268700	1.83466700	0.45294800
C	-5.97872400	-1.53944500	-1.60487600
C	-4.58089800	-1.16628600	-1.10508300
O	-3.59240400	-1.72768800	-1.68338700
O	-4.52504000	-0.33104400	-0.15087300
C	-1.27740500	-4.49517000	-0.31784100
O	-0.77081900	-4.21992000	0.78902600
O	-1.66408800	-3.65491000	-1.22783000
Ca	0.19702700	-2.18188200	1.57304800

O	-2.09591300	-1.65853600	0.66691000
O	0.11835200	-1.67933400	-0.73150200
O	-1.90607300	0.22610700	-0.99189900
O	2.61452200	-0.59043400	-1.01138900
O	1.32005600	-0.13801900	1.03184800
O	-0.79971400	0.85446800	-0.30708700
Mn	-2.65269300	0.05562100	0.82594600
Mn	-1.64006000	-1.71658000	-1.10499000
Mn	0.92999100	0.01204400	-0.74595100
Mn	3.15183200	-0.32471400	0.75610100
O	4.75048100	0.52051400	-2.52004200
O	2.16402200	-3.25638300	0.39410500
O	3.43864100	-0.56752000	2.64606800
O	1.88453100	-2.53727700	3.31578600
C	-0.01130000	-0.60607600	-4.95190000
C	-0.25263300	-0.67182600	-3.44288100
O	-1.15876600	-1.47847200	-3.04089000
O	0.48156000	0.08680700	-2.73995500
H	7.49562200	-2.35948100	1.04720500
H	7.10980200	-3.07500100	-0.51012000
H	-2.96797400	0.27583100	5.32081500
H	-3.90320100	-1.13255500	4.85977900
H	-0.32415900	4.98115900	2.25242800
H	-1.81478900	5.58374600	2.96384600
H	-2.19008700	2.52705300	2.88139300
H	-3.39780300	2.57795600	-1.14592700
H	2.35733600	4.41481500	-2.44522000

H	0.88165300	4.09934000	-1.54379100
H	3.74501000	4.20837700	-0.42243500
H	2.33655300	4.33286300	0.63312200
H	1.27308600	6.56216100	0.73537000
H	-6.46543300	-2.07382100	-0.77063300
H	-6.54205000	-0.59924400	-1.71810100
H	1.01292900	-0.98398600	-5.11017700
H	0.02765200	0.46149700	-5.22075700
H	3.89428800	0.12834000	-2.21529100
H	5.24413700	0.52982100	-1.67948700
H	3.15656900	-3.16082200	0.50749500
H	1.97571200	-2.81624000	-0.46202500
H	4.38031500	-0.76142900	2.79642100
H	2.52712100	-1.74286400	3.13836900
H	2.40433300	-3.30648800	3.02314200
C	-1.01643000	-1.36740800	-5.81422800
H	-2.03564400	-0.97949300	-5.67520400
H	-0.75533300	-1.27669700	-6.88039000
H	-1.03631400	-2.43354100	-5.55083500
C	7.68225800	-0.96837800	-0.61715600
H	7.58545700	-0.05085700	-0.02024500
H	8.75459400	-1.16832500	-0.77311500
H	7.22577900	-0.77642300	-1.59923700
C	-2.11735600	-1.60851900	6.02742200
H	-1.08795400	-1.24698300	6.15598400
H	-2.62695500	-1.56910900	7.00283800
H	-2.05164500	-2.65914800	5.71289900

C	-1.45761900	-5.95662000	-0.70795000
H	-1.73099600	-6.54532900	0.17736800
H	-2.20557100	-6.07331100	-1.50136600
H	-0.49073400	-6.33253600	-1.07710000
C	-6.01824800	-2.37651100	-2.88236500
H	-7.05762700	-2.61858100	-3.15482800
H	-5.55797700	-1.83958200	-3.72381000
H	-5.46240900	-3.31502500	-2.75569200
C	1.64243900	6.11015400	-1.32893200
H	2.59902300	6.64740200	-1.22707800
H	1.12492400	6.50316700	-2.21516500
C	-1.24306900	6.61622800	1.16044300
H	-2.24590600	7.01083100	0.94201600
H	-0.71375300	7.37470200	1.75982100
N	-2.53764600	4.13959000	0.06896400
H	-2.35319700	4.88159800	-0.62311400

TS for S_3-state model in the peroxo state with O3-O6 bond at ~1.80Å

with Mn4-H_2O, -OH, charge -1. S=1 and His337-Nδ-H* configuration

* Lack of the H-bond between the His337-Nε-H and O3 makes His337 drift away from our unrestrained cluster OEC model. Thus, His337 was removed from the model for this set of calculations.

C	6.88715100	1.91429300	1.16774600
C	5.44639100	1.39326900	1.15470000
O	5.22238500	0.25287000	1.62173700
O	4.54698600	2.19863700	0.68634700
C	-2.16769600	-2.52327400	4.89445300
C	-1.64608100	-1.86956400	3.61197800

O	-0.42085100	-1.68946700	3.45357300
O	-2.57931700	-1.53728500	2.77627500
C	-4.06356600	5.12272400	-0.50006400
O	-4.92604500	4.55971100	-1.19530000
C	-3.69096000	4.40660400	1.96115200
C	-3.84731800	2.95342100	1.60992500
C	-3.13487100	1.85573600	2.03804400
C	-4.56177900	1.11850800	0.57365300
N	-3.58425000	0.72543000	1.38412500
N	-2.84658000	5.48878000	-0.97748100
C	-1.34412200	3.91879000	-2.22435600
C	-0.01175400	4.28237500	-1.55757300
C	0.83449600	3.07243900	-1.13674000
O	0.55068400	1.97053900	-1.71336200
O	1.71097700	3.27381700	-0.25164900
C	-4.41529700	-3.54273200	-1.88692700
C	-3.30465200	-2.73939000	-1.19947500
O	-2.13635300	-2.84845300	-1.69188400
O	-3.66384300	-2.04413500	-0.19798100
C	0.76720600	-4.57380000	0.26531400
O	1.14481700	-3.93412100	1.27244500
O	0.17482600	-4.09794300	-0.78124900
Ca	1.23770000	-1.59400200	1.72435800
O	-0.89529500	-1.96685200	0.61170500
O	1.38404600	-1.65994000	-0.67836200
O	-0.66279600	-0.45613200	-1.49427700

O	2.88525100	0.67775700	-0.98315200
O	1.06493400	0.57592300	0.64807500
O	-1.86734600	0.23970800	-0.35211900
Mn	-2.27957500	-0.88761000	0.93541400
Mn	-0.30937900	-2.20827900	-1.06918700
Mn	1.17335300	0.15301500	-1.12037200
Mn	2.64967300	1.55674100	0.64198800
O	4.61324300	2.44782300	-2.42189100
O	3.60055900	-1.85975300	0.94325000
O	2.43003400	2.01163700	2.49732600
O	2.57697400	-0.42707700	3.42255400
C	1.43489200	-1.80050400	-5.00474900
C	0.99753300	-1.50323300	-3.57062800
O	0.27284500	-2.38084700	-2.99160900
O	1.40812700	-0.40502900	-3.08527300
H	7.11674700	2.14761500	2.22295100
H	7.53198300	1.06077000	0.90795200
H	-2.88854100	-1.82011900	5.34456900
H	-2.76748000	-3.39592000	4.58671800
H	-2.61127500	4.60666000	2.04983700
H	-4.11407400	4.60758600	2.96105100
H	-2.30454100	1.81298900	2.73567000
H	-5.07991400	0.47960000	-0.13323300
H	-1.15739300	3.55498800	-3.24615800
H	-1.80845700	3.07670200	-1.68899500
H	0.60767000	4.90678500	-2.22457900

H	-0.15775600	4.87307400	-0.63789800
H	-2.18076600	5.88098300	-0.31715000
H	-4.82160400	-4.22445000	-1.12058300
H	-5.22990200	-2.83381200	-2.10947400
H	2.53527300	-1.87208000	-4.97924700
H	1.21403500	-0.89775900	-5.59632600
H	3.98467500	1.72368300	-2.18657300
H	4.74510500	2.85327400	-1.54475600
H	4.20451400	-1.07844700	1.06230900
H	3.29965600	-1.81357500	0.01033300
H	3.19659900	2.54296100	2.77640900
H	2.48189900	0.56842600	3.18441300
H	3.51924400	-0.58653400	3.22512200
C	0.81775000	-3.04892100	-5.63263700
H	-0.27744500	-2.97035900	-5.68039100
H	1.19884200	-3.19279100	-6.65583400
H	1.05353600	-3.94594200	-5.04424500
C	7.17471100	3.12184200	0.27526500
H	6.53485800	3.97413900	0.54318600
H	8.22634900	3.43547900	0.37255000
H	6.98723300	2.88365300	-0.78187200
C	-1.08055600	-2.91902300	5.89129000
H	-0.48576600	-2.04671800	6.19556000
H	-1.52502600	-3.37049200	6.79225100
H	-0.38453300	-3.64472200	5.44831200
C	1.03336900	-6.07423700	0.20894100

H	0.90593100	-6.51241600	1.20756100
H	0.38169700	-6.57059800	-0.52021500
H	2.08105600	-6.22725300	-0.09349000
C	-3.99229600	-4.31170500	-3.13700300
H	-4.84533200	-4.86889900	-3.55584200
H	-3.61006300	-3.63113000	-3.91027100
H	-3.18814400	-5.02408100	-2.90885800
C	-2.34068000	5.08499100	-2.29576900
H	-1.88569600	5.96846900	-2.77494900
H	-3.22514200	4.79885100	-2.88007400
C	-4.33140200	5.41809900	0.97847200
H	-5.42480900	5.41666100	1.09144600
H	-3.97884400	6.42814200	1.24294900
N	-4.77016500	2.45280500	0.69870900
H	-5.29475500	3.03950400	0.03767600

References

1. Becke, A.D., *DENSITY-FUNCTIONAL EXCHANGE-ENERGY APPROXIMATION WITH CORRECT ASYMPTOTIC-BEHAVIOR.* Physical Review A, 1988. **38**(6): p. 3098-3100.
2. Perdew, J.P. and W. Yue, *Accurate and simple density functional for the electronic exchange energy: Generalized gradient approximation.* Physical Review B, 1986. **33**(12): p. 8800-8802.
3. Weigend, F. and R. Ahlrichs, *Balanced basis sets of split valence, triple zeta valence and quadruple zeta valence quality for H to Rn: Design and assessment of accuracy.* Physical Chemistry Chemical Physics, 2005. **7**(18): p. 3297-3305.
4. Pushkar, Y., K.M. Davis, and M. Palenik, *Model of the Oxygen Evolving Complex Which is Highly Predisposed to O–O Bond Formation.* Journal of Physical Chemistry Letters, 2018. **9**: p. 3524-3531.
5. Pushkar, Y., et al., *Early Binding of Substrate Oxygen Is Responsible for a Spectroscopically Distinct S2 State in Photosystem II.* The Journal of Physical Chemistry Letters, 2019. **10**(17): p. 5284-5291.

6. Bury, G. and Y. Pushkar, *Insights from Ca2+→Sr2+ substitution on the mechanism of O-O bond formation in photosystem II.* Photosynthesis Research, 2024. **162**(2): p. 331-351.
7. Davis, K.M., et al., *Rapid Evolution of the Photosystem II Electronic Structure During Water Splitting.* 2015: p. https://arxiv.org/abs/1506.08862 (accessed May 17, 2017).
8. Davis, K.M. and Y.N. Pushkar, *Structure of the Oxygen Evolving Complex of Photosystem II at Room Temperature.* Journal of Physical Chemistry B, 2015. **119**(8): p. 3492-3498.
9. Davis, K.M., et al., *X-ray Emission Spectroscopy of Mn Coordination Complexes Toward Interpreting the Electronic Structure of the Oxygen-Evolving Complex of Photosystem II.* The Journal of Physical Chemistry C, 2016. **120**(6): p. 3326-3333.
10. Jensen, S.C., et al., *X-ray Emission Spectroscopy of Biomimetic Mn Coordination Complexes.* Journal of Physical Chemistry Letters, 2017. **8**(12): p. 2584-2589.
11. Siegbahn, P.E.M., *O-O bond formation in the S4 state of the oxygen-evolving complex in photosystem II.* Chemistry-a European Journal, 2006. **12**(36): p. 9217-9227.
